# A statistical framework for differential network analysis from microarray data

**DOI:** 10.1186/1471-2105-11-95

**Published:** 2010-02-19

**Authors:** Ryan Gill, Somnath Datta, Susmita Datta

**Affiliations:** 1 Department of Mathematics, University of Louisville, Louisville, KY 40292, USA; 2 Department of Bioinformatics and Biostatistics, University of Louisville, Louisville, KY 40202, USA

## Abstract

**Background:**

It has been long well known that genes do not act alone; rather groups of genes act in consort during a biological process. Consequently, the expression levels of genes are dependent on each other. Experimental techniques to detect such interacting pairs of genes have been in place for quite some time. With the advent of microarray technology, newer computational techniques to detect such interaction or association between gene expressions are being proposed which lead to an association network. While most microarray analyses look for genes that are differentially expressed, it is of potentially greater significance to identify how entire association network structures change between two or more biological settings, say normal versus diseased cell types.

**Results:**

We provide a recipe for conducting a differential analysis of networks constructed from microarray data under two experimental settings. At the core of our approach lies a connectivity score that represents the strength of genetic association or interaction between two genes. We use this score to propose formal statistical tests for each of following queries: (i) whether the overall modular structures of the two networks are different, (ii) whether the connectivity of a particular set of "interesting genes" has changed between the two networks, and (iii) whether the connectivity of a given single gene has changed between the two networks. A number of examples of this score is provided. We carried out our method on two types of simulated data: Gaussian networks and networks based on differential equations. We show that, for appropriate choices of the connectivity scores and tuning parameters, our method works well on simulated data. We also analyze a real data set involving normal versus heavy mice and identify an interesting set of genes that may play key roles in obesity.

**Conclusions:**

Examining changes in network structure can provide valuable information about the underlying biochemical pathways. Differential network analysis with appropriate connectivity scores is a useful tool in exploring changes in network structures under different biological conditions. An R package of our tests can be downloaded from the supplementary website http://www.somnathdatta.org/Supp/DNA.

## Background

Construction of biological networks (gene-gene, protein-protein, gene-protein, etc.) has been of considerable interest amongst computational biologists as is evident by a fast growing literature [1]. Often, network construction via computational methods is considered to be a faster and more viable alternative to experimental methods, especially, for high throughput studies. It can be argued that, in many genomic studies, it is of even greater interest to see how the network of connected gene pairs change from one experimental condition to another since such changes may offer an important clue regarding an underlying biological process such as identification of pathways that correspond to such a change.

Whereas a variety of network construction methods now exist, methodologies for a differential network analysis are few and far between. It is therefore the purpose of this paper to introduce a formal statistical methodology to detect significant changes in two biological networks. We describe and study our methods in the context of gene-gene interaction networks although it is conceivable that the methods can be easily adapted to other types of biological networks. Specifically, we are interested in statistical tests for answering the following questions related to networks constructed using the same set of genes under two experimental conditions: (i) whether the overall modular structures of the two networks are different, (ii) whether the connectivity of a particular set of "interesting genes" has changed between the two networks, and (iii) whether the connectivity of a given single gene has changed between the two networks. The building blocks of all our statistical tests are the set of scores that measure the strength of association/interaction between gene pairs in the two networks. We provide examples of a number of measures of gene-gene association/interaction such as correlation, partial correlation, mutual information, posterior probabilities and so on. Another measure that is heavily used in this paper is based on a partial least squares [2-5] modeling of one gene's expression on the remaining genes. These scores were introduced in our earlier paper [6] on genetic network reconstruction.

An early attempt to study how pairwise correlation between genes in two plants change was presented in [7]. A differential network analysis using liver gene expression data in normal versus heavy mice was performed in [8] in an attempt to identify the underlying genetic drivers and pathways, and they also proposed a test for differential connectivity of a single gene. A side by side comparison of gene expression networks for normal versus CFS (Chronic Fatigue Syndrome) patients was performed in [9] through a visual analysis and detected change in connectivity of certain node genes although they did not carry out any statistical significance tests. Their association scores were based on a mutual information criterion [10]. Finally, [11] fit separate structural equations to the two sets of gene expressions and tested the null hypothesis of equality of the coefficients in the two models as an indication of equality in the overall network structures.

In the Methods section, we describe an approach of measuring association/interaction using connectivity scores, and we primarily use scores based on PLS [6]. We also describe how to identify modules and hub genes from these scores in an unsupervised manner. Then we formulate the three test statistics to inspect various aspects of how the two networks are different. Unlike previous approaches, this approach offers a formal statistical test using each notion of differential connectivity. Our simulation results are reported in the Results section. We simulate from two different types of models where we know some form of the ground truth. In the Results section, we also reanalyze a data set on a mouse obesity study.

## Results and discussion

We investigate the performance of our testing procedures in a number of simulated data sets as well as one real data set. As can be seen from these studies, the proposed statistical tests are effective in detecting differences between the network structures.

### Simulated data

We use two types of simulation models to generate data. The first approach uses partial differential equations to model expression levels, and one could generate networks of various structures and complexities that are presumably quite realistic. The second simulation model generates a simple Gaussian network where (transformed) gene expressions are generated from a multivariate normal distribution. By selecting the variance-covariance matrix, we can induce various types of association/interaction amongst the genes; another advantage of this model is that replicated data sets can be generated with the same network structure so that statistical properties of our tests (i.e., size and power) can be computed empirically.

#### Differential equation based networks

**Figure 1:**
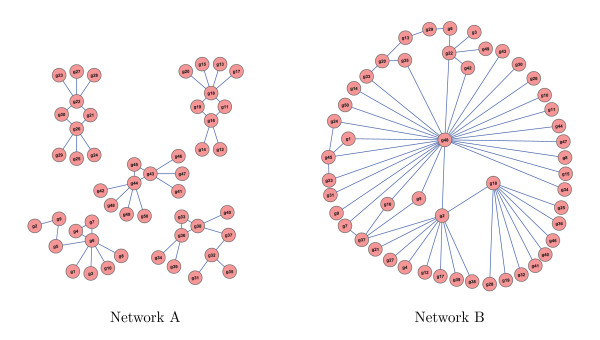
**Two simulated networks**. Gene expression data on fifty samples on fifty genes in two networks were simulated using SynTReN. Network A had five modules whereas Network B had one.

The SynTReN software developed by [12] simulates biological networks with known underlying structures based on existing biological subnetworks and are modeled with Michaelis-Menten and Hill kinetic equations. This software was used to generate two networks with *N*_*i *_= 50 samples each and *p *= 50 genes. The first network (network A) consists of five separate modules with ten genes each while the second network (network B) has a single module with all 50 genes. The software allows the user to specify several tuning parameters which control the noise and complexity in each generated network; all probability parameters were set to 0.05 for both the treatment and control networks. The two networks are illustrated in Figure 1 using the Cytoscape software [13].

We consider testing for differential modular structures in the two networks as described in the Methods section using PLS connectivity scores. Clearly, the performance of the method will depend on the choice of the minimum module size *m *and ϵ, which is a user selectable parameter threshold on the connectivity scores to determine if there should be an edge between two nodes (genes) in a network. If ϵ is too large, then the method will find very few interactions between genes and therefore very few (if any) modules; if ϵ is too small, then the method will find too many interactions between the genes and every gene will be in the same large module. As *m *increases, the number of modules *J*_1 _and *J*_2 _decreases. Consequently, we performed a sensitivity analysis with respect to the tuning parameters *m *and ϵ. The p-values were computed using *P *= 1000 random permutations each.

In this simulated example, there is a statistically significant difference between the modular structures for a wide range of values of *m *and ϵ based on the test for different modular structures in the Methods section. Table 1 shows the results for the minimum module size of *m *= 5 with various choices of ϵ, and presents the value of the test statistic  and its corresponding p-value. The results for other values of *m *between 1 and 8 are very similar and available on the supplementary website [14].

**Table 1:** Tests for differential modular structure in the two networks created by SynTReN software.

ϵ		p-value
.20	.641	.000
.25	.862	.000
.30	.910	.000
.35	.919	.001
.40	.965	.003

#### Gaussian networks

We conducted a simulation study based on two networks (treatment and control) each generated from the multivariate Gaussian distribution with a zero mean vector. Under this setup, we investigate the statistical power of the test for differentially connectivity for each of the genes described in the Methods section.

We report the results for two network settings, one with *p *= 20 genes and another with *p *= 100 genes. Additional results for other setups, including PLS scores, are available on a supplementary website [14]. For each Monte-Carlo sample, the p-value of each test is based on 1000 random permutations. Since each such calculation is based on Monte-Carlo replications of the original samples whereas the observed level of significance (p-value) for each original sample is based on another level of Monte-Carlo iteration, the total computational demand is fairly substantial. As a result we base our calculations on 1000 Monte-Carlo iterates for the 20-gene and 100-gene networks.

The covariance matrix of the control network is taken to be the identity matrix. This signifies a hypothetical situation where none of the genes is interacting with one another. The diagonal elements of the covariance matrix of the treatment network are 1, the off-diagonal elements of the first 10 genes are *ρ *or -*ρ *depending on whether the sum of the respective indices are even or odd, and the remaining off-diagonal elements are 0. Three values of *ρ *were used where larger *ρ *indicates higher association so that we may expect the power of our test to increase with *ρ*. Thus, the first 10 genes are the "important" genes whose connectivity is present in the treatment network but not in the control network; the remaining genes are "unimportant" which behave independently in both networks. In this study, we selected two sample sizes, *n *= 50 and 200. A reasonable test should have an increasing power function with the increasing sample size.

In each setting, we compute the following quantities:

(i) Sensitivity: This is computed by proportion amongst the "important" genes that were declared to be significantly differentially connected.

(ii) Specificity: This is computed by proportion amongst the "unimportant" genes that were declared to be not significantly differentially connected.

(iii) True discovery rate (TDR): This is computed by proportion amongst genes declared significantly differentially connected that were amongst the "important" genes.

(iv) True non-discovery rate (TNR): This is computed by proportion amongst genes declared to be not significantly differentially connected that were amongst the "unimportant" genes.

High values of each of these measures indicate good performance of the testing procedure in some aspect. Note that the sensitivity is the same as the average power; that is, it is the proportion of Monte-Carlo samples for each given "important" gene in which it is declared to be significantly differentially connected averaged across all ten "important" genes. Similarly, one minus the specificity is the average size.

Here we report the results for our tests for the correlation scores. Since it is a Gaussian network that was constructed using various degrees of correlation between the dependent genes, the results using the sample correlation are the easiest to interpret and most natural. The supplementary website lists the results using other scores. Results for *p *= 20 are summarized in Table 2 whereas those for *p *= 100 are listed in Table 3. The nominal size for all tests was set at *α *= 5% which means a gene is declared to be differentially connected if its permutation based p-value is less than 0.05. The results for *α *= 10% are provided in the supplementary website [14].

**Table 2:** Empirically estimated performance measures for the tests of differential connectivity of single genes using correlation scores applied at a targeted nominal level of 5%.

*n*	*ρ*	Sensitivity		Specificity		TDR		TNR	
		unadjusted	adjusted	unadjusted	adjusted	unadjusted	adjusted	unadjusted	adjusted
50	.5	.990	.893	.949	.970	.951	.970	.989	.982
200		1	1	.946	.970	.949	.971	1	1
									
50	.7	1	1	.948	.969	.951	.970	1	1
200		1	1	.950	.973	.952	.973	1	1
									
50	.9	1	1	.946	.969	.948	.970	1	1
200		1	1	.948	.968	.950	.969	1	1

The measures are reported based on both unadjusted p-values and p-values adjusted based on the BH multiplicity correction. The number of "important" genes was 10 and the total number of genes was 20.

**Table 3:** Empirically estimated performance measures for the tests of differential connectivity of single genes using correlation scores applied at a targeted nominal level of 5%.

*n*	*ρ*	Sensitivity		Specificity		TDR		TNR	
		unadjusted	adjusted	unadjusted	adjusted	unadjusted	adjusted	unadjusted	adjusted
50	.5	.760	.340	.947	.996	.616	.913	.973	.931
200		1	.999	.949	.994	.687	.946	1	1
									
50	.7	.986	.862	.948	.993	.676	.935	.998	.985
200		1	1	.949	.993	.685	.943	1	1
									
50	.9	1	.996	.946	.992	.675	.936	1	1
200		1	1	.949	.994	.684	.948	1	1

The measures are reported based on both unadjusted p-values and p-values adjusted based on the BH multiplicity correction. The number of "important" genes was 10 and the total number of genes was 100.

For *p *= 20, the sensitivity of all the tests performed together is close to 1. In order to account for simultaneous testing of multiple hypotheses, we also considered the standard Benjamini-Hochberg(BH) [15] adjusted p-values to declare significance. We also attempted other relatively recent multiple hypotheses adjustment procedures. These include the local FDR due to [16], the q-value due to [17], and the fdrtool due to [18]. Their performances varied across the different measures but overall, none of them seem to do better than the tests without any p-value adjustments. Consequently, these are not reported in the tables.

For *p *= 100, the sensitivity increased as *ρ *increased and was lower for the BH adjustments. The procedures have high specificity with the BH adjustment. The TNR is also close to 1 and the unadjusted TDR ranges between 60%-70% which suggest that some unimportant genes were deemed to be differentially connected by the procedures which is not unexpected since only 10% of the genes were truly important. The BH adjustment improved the TDR to between 90%-95%.

In this simulation experiment, we were aware of the identity of the important genes. We also investigated the performance of the test for differential connectivity of a class of genes described in the Methods section. In each case, the power for detecting the class of important genes is 1 when using pairwise correlations. Full results are presented in the supplementary website [14].

The analysis for the Gaussian simulations is based on the statistical tests described in the Methods section. While it is certainly possible to use regularized statistical tests for the Gaussian model based on appropriate asymptotic theory, we only present the Gaussian model as a simple model for which we can easily conduct a simulation study. The more general statistical tests described in the Methods section are not only applicable to the Gaussian model, but also to more complex models which are more appealing from a biological perspective.

### Real data

We illustrate our methodology using a real data set.

#### Mouse data

We apply our tests described in the Methods section to a subset of microarray expression data obtained from liver tissue of female mice and corresponding clinical traits for the mice that was analyzed previously by [8]. The full data set consists of 3421 genes and 135 mice. The data set was further reduced by removing genes and mice with missing values. For the differential analysis, we selected two networks of mice. The first network consisted of the 50 heaviest mice with weights greater than 40.5. The second network consisted of the 50 leanest mice with weights less than 36.9. We worked with a filtered collection of genes  based on univariate regressions of mouse weights on each individual gene's expressions using all mice; we chose the 314 genes with z-scores greater than 5.

Using PLS connectivity scores and the test for differential structures (with a minimum module size of *m *= 5 and threshold connectivity score of ϵ = 0.5), the value of the test statistic is  = .976 with a p-value of *p*(ℱ) = 0.033 based on 1000 random permutations; thus the modular structures of the two networks are significantly different at a 5% level. The module structures of the two networks are illustrated in the Supplementary Material website using the Cytoscape software [13]. In addition we performed a sensitivity analysis with respect to varying ϵ and found that, for any moderate choice of ϵ, the modular structures are statistically significantly different. The complete results for ϵ ∈ {-0.35, 0.40, 0.45, 0.50, 0.55} are presented in the Supplementary Material website [14].

Next, using the test for differential connectivity of individual genes, we found 56 genes which are significant at level 0.05 without any multiplicity correction. The gene names, values of the test statistic, and the corresponding p-values for the 20 most differentially connected genes are listed in Table 4. A complete list is given on the Supplementary Material website. Below, we give a brief commentary for the biological functions of a select few genes which we mined using the Entrez Gene tool [19].

**Table 4:** The 20 most differentially connected genes based on the test for differential connectivity between the lean and heavy mice networks.

Gene	*d*	p-value	Gene	*d*	p-value
*Anxa2*	0.118	0.000	*Spp1*	0.232	0.000
*Anxa5*	0.119	0.000	*9430028I06Rik*	0.153	0.000
*Apom*	0.186	0.000	*AA960558*	0.153	0.001
*F*7	0.122	0.000	*Map4k4*	0.145	0.001
*Igfbp7*	0.157	0.000	*Proz*	0.126	0.001
*Itih1*	0.149	0.000	*2310046G15Rik*	0.158	0.001
*Kng2*	0.168	0.000	*Erbb3*	0.167	0.003
*Scnn1a*	0.149	0.000	*Ppic*	0.097	0.003
*Slc22a7*	0.154	0.000	*Tuba1*	0.132	0.003
*Slc43a1*	0.162	0.000	*Igfbp2*	0.182	0.004

The first two genes on this list, *Anxa2 *and *Anxa5*, encode members of the annexin family. Members of this calcium-dependent phospholipid-binding protein family play a role in the regulation of cellular growth and in signal transduction pathways. This protein functions as an autocrine factor which heightens osteoclast formation and bone resorption.

Apolipoprotein M, also known as *APOM*, is a human gene. The protein encoded by this gene is an apolipoprotein and member of the lipocalin protein family. It is found associated with high density lipoproteins and to a lesser extent with low density lipoproteins and triglyceride-rich lipoproteins. The encoded protein is secreted through the plasma membrane but remains membrane-bound, where it is involved in lipid transport.

The gene *F7 *initiates the extrinsic pathway of blood coagulation. In the literature, this gene has been tested for association to various diseases including blood coagulation disorders, hepatocellular carcinoma, cardiovascular diseases, cerebral infarction, coronary disease, and diabetic angiopathies.

The fifth gene on this list, *Igfbp7*, has been tested in the literature for association to various form of neoplasms. It has been proposed to participate in processes such as negative regulation of cell proliferation and regulation of cell growth.

**Figure 2:**
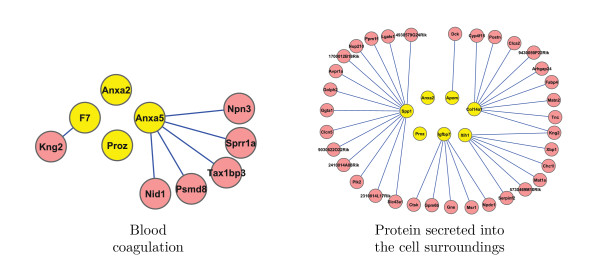
**Network structures for the mouse data**. Connectivity for the heavy mice network of the gene clusters in Table 5 (displayed for gene pairs with || ≥ 0.5). In contrast, there were no connections between these gene pairs for the lean mice network (not shown here).

We used DAVID [20] functional clustering with the genes in Table 4. The following two functional clusters reported in Table 5 were identified on the basis of enrichment scores (as performed by DAVID using Fisher's exact test). We also performed (post-hoc) the test for differential connectivity of these functional classes in the two networks of lean versus heavy mice. The corresponding p-values were less than 10^-3 ^(the last column of Table 5). Figure 2 illustrates the connectivity for the heavy mice network of the two functional gene clusters for gene pairs with scores which exceed 0.5 in magnitude. For the lean mice network, there were no such connections between these gene pairs.

**Table 5:** Functional clustering of differentially connected genes.

Cluster Description	Genes	p-value
Blood coagulation	*Anxa2*, *Anxa5*, *F*7, *Proz*	0.000
Protein secreted into the cell surroundings	*APOM*, *Anxa2*, *Spp1*, *Igfbp7*, *Itih1*, *Proz*, *Col14a1*	0.000

## Conclusions

Studying how the network structure changes between two conditions (e.g., two stages of a biological process) offers important clues about the underlying biochemical pathways. Differential network analysis, as we call it, provides formal statistical tests to undertake such an exploratory investigation. This is often done in conjunction with a differential gene expression analysis and offers a deeper understanding than that obtained by a list of genes that are differentially expressed between the two conditions. Indeed such a list can be used as a filtering or selection step where the network structures of these genes are further explored under the two conditions. While the real data example presented in this paper is a fixed-time experiment, the methods could be used to examine whether a network is differentially expressed over two distinct time periods. However, analyzing dynamic networks with multiple conditions is a more complicated topic for future investigation.

We explore the use of connectivity scores in the construction of measures of the strength of a relationship between a pair of genes in a network and how this strength changes from one biological condition to another. Simulation investigations of our method are carried out using appropriate sets of scores. Although the formulas for the test statistics can easily be adapted to incorporate other measures of association or interaction, it is important to choose an appropriate measure of connectivity. PLS scores tend to work well in most circumstances.

While the precise calibration of the statistical level is problematic, the resulting methods using an approximate level of p-value control results in reasonable performance (in terms of various measures, as demonstrated empirically). Also, the utility of these methods for exploratory analysis is well demonstrated by the real data application.

There is scope of further theoretical work towards obtaining a better approximation to the statistical null distribution. Development of appropriate global error rate control statistical adjustments is another interesting problem in this regard. Essentially all the existing procedures rely on the independence (or some form of weak dependence) of multiple statistical tests which is not satisfied for testing change of interaction scores of pairs of genes. These issues will be investigated elsewhere.

## Methods

Some notations are necessary to describe our statistical tests. We assume that two microarray studies are conducted on the same set of genes but under two different biological conditions. Here the term "biological condition" is very generic and depending on the application may correspond to, for example, subject type (e.g., male versus female), tissue type (normal versus cancerous) or time index in a time course experiment. We assume that the data (normalized and often log transformed gene expression values) for each study can be represented by an *N *× *p *matrix *X *where *N *is the number of subjects in the study and *p *is the number of genes (or more appropriately, probes) in the study. Typically, some form of scores are constructed from the expression matrix *X *for each pair of genes to measure the interaction between them, and a network is constructed by connecting the pairs for which the corresponding score exceeds a threshold.

### Connectivity score between a pair of genes

Each of the statistical tests described in this section are based on a connectivity score  between the *i*th and *k*th gene derived from *X*. Let *x*_*i *_be the (centered and scaled) expression vector for the *i*th gene. Here we describe some choices of the connectivity scores that could be used for conducting our statistical tests. All these measures of association/interaction between genes have been previously proposed in the literature for reconstruction of genetic networks.

#### Correlation

A widely used simple measure of strength of the association between two genes is the Pearson correlation coefficient. The correlation between gene *i *and gene *k *is given by

This gives the coefficient of a simple linear regression model of one gene's expression values on the other, given that both are standardized. For a more detailed discussion, see [21] and the references therein.

#### Partial correlation

Partial correlation (PC) based scores for network construction were proposed by [22]. The partial correlations are related to the inverse of the standard correlation matrix *P *and can be computed using the following relationships

and

The authors also proposed a modification when the covariance matrix is not positive definite (and thus not invertible) which is the case when *N *<*p*. They used the Moore-Penrose pseudoinverse followed by bagging. However, in a later paper [23], they proposed a covariance shrinkage estimator given by

where  denotes the estimate of the covariance matrix *P*, *T *denotes the constrained shrinkage target covariance matrix of a lower complexity (assuming some form of structure such as equal variances, constant correlations etc.), and *λ *is the shrinkage coefficient which balances the bias-variance tradeoff of the two estimates  (characterized by a relatively large variance) and *T *(biased due to imposed constraints).

#### Partial least squares based scores

For general complex data sets, the association/interaction scores introduced in our earlier work [6] tend to work well. The basis for these scores is a set of partial least squares (PLS) fits of each gene's expression vector, on that of the remaining genes, such that(1)

where *v*, denoting the number of PLS terms , is a user selectable tuning parameter and the PLS components  are linear combinations of *x*_1_,⋯, *x*_*i*-1_, *x*_*i*+1_,⋯, *x*_*p *_that are algorithmically obtained as follows:

(i) Set ℓ = 1 and *X*^(1) ^= [*x*_1_,⋯, *x*_*i*-1_, *x*_*i*+1_,⋯, *x*_*p*_].

(ii) Compute

where

(iii) Increase ℓ to ℓ + 1, compute the deflated design matrix

and while ℓ ≤ *v*, go to Step (i).

It is argued in [6] that

is an appropriate (weighted) measure of total association/interaction between the pair of genes *i *and *k*, where  are the least squares estimates of the coefficients in model (1). For further details on these scores, see [6]. For more background on partial least squares regression, we refer the readers to [2-5] and the references therein. An alternative way to define PLS scores based on the product of PLS regression coefficients is described in [24].

When *N *and *p *are large, the statistical tests based on PLS scores are computationally intense. For the mouse data described in the Results and discussion section, the computing times for each of the tests using PLS scores and 1000 permutations are approximately 40 minutes on a Linux machine with Intel Xeon 3.20 GHz processors. Each of the statistical tests described in this section are implemented in an R [25] package freely available on the supplementary website [14].

### Modules of genes

Often biological networks have a modular structure where a cluster of genes is connected by short paths whereas genes that belong to different clusters have no connectivity, indicating no (or weak) association/interaction between them. In an unsupervised study, one of the goals of a network analysis is to identify all such modules. These are mostly accomplished through visual means. However, a mathematically convenient definition of a module after a network has been constructed is provided here. Such an approach is useful in constructing a test for investigating whether the overall modular structures in two networks are different.

To this end, we use the following mathematical definition of a module of genes in reference to an association/interaction network. We like to point out that the term "module" has been used in the past by different authors in different contexts (see [26] and the references therein). In our definition, the minimum size parameter *m *and the threshold connectivity parameter ϵ are user selectable making this approach suitable for an exploratory analysis. With these two parameters in place, a collection of genes ℱ will be called a module if *f *= |ℱ|, the cardinality of ℱ, is at least *m *and, given any two genes *f*_1 _and *f*_2 _in ℱ, they are connected by a path of genes in ℱ, *f*_1 _= *g*_1_,⋯, *g*_*k *_= *f*_2_, for some *k *≥ 2, such that the association/interaction score of each pair on the path is at least ϵ, i.e.,  ≥ ϵ, for all 1 ≤ *j *≤ *k *- 1. Moreover, such a set has to be a maximal collection so that, for any gene *g *∉ ℱ, |*s*_*gf*_| < ϵ, for all *f *in ℱ.

### Testing for differential modular structures in two networks

Suppose two networks have been constructed, say, using the control (*X*_1_) and the treatment (*X*_2_) samples, respectively. Given a selection of the two tuning parameters *m *and ϵ, we could identify the collection of all modules (as defined above) in the two networks. Let  be all the distinct modules of size at least *m *and connectivity ϵ in network *k*, for *k *= 1, 2. Let  be the collection of all genes that were present in some module in both networks. In other words, .

Given a gene *g *∈ , let ℱ_*kj*(*g*) _be the module in network *k *that contains gene *g*, for *k *= 1, 2. The following proportion of non overlap statistics captures the amount of differentiation within the modular structures in the two network:

where an empty sum (e.g., when  = *ϕ*) is to be interpreted as 0. Note that it lies between 0 and 1 where 0 indicates identical modular structure in the two networks and 1 indicates that the modules in the two networks have nothing in common.

Also note that the modules according to our definition are necessarily disjoint and hence the test statistic  is well defined. If one uses alternative definitions of modules in which a gene is allowed to belong to multiple modules, the statistic needs to be modified. As for example, we could replace the summand in  by an average of similar quantities over the pairs of modules containing gene *g*. Further consideration of alternative definitions of modules is beyond the scope of this paper.

For controlling the type-1 statistical error rate, one needs to compute the p-value using the following permutation scheme. Let *X*_*k *_be the (*N*_*k *_× *p*) matrix of expression values of the *N*_*k *_samples (replicates) of *p *genes, *p *= ||, for *k *= 1, 2. Let  be the (*N*_1 _+ *N*_2_) × *p *matrix in which the first *N*_1 _rows of  are the *N*_1 _rows of *X*_1 _and the last *N*_2 _rows of  are the *N*_2 _rows of *X*_2_. Permute the rows of  using a permutation *π *to get the permuted matrix , and let  be the first *N*_1 _rows of  and  be the remaining *N*_2 _rows of . For each permutation *π*, compute the collection of pairwise scores denoted , *k *= 1, 2, using the  and  data respectively and compute the test statistic on the permuted data as

where ℱ_*kj*_(*π*) are the distinct modules in the two networks based on the permuted data, , and so on. In other words, we permute the labels of the samples and perform our analysis again for each permutation.

After computing the MDA statistic corresponding to *P *permutations selected at random from the collection of all permutations, we can obtain an approximate *p*-value by computing

where the sum is taken over the *P *random permutations *π*. Under the null hypothesis that the modular structure of the two networks is the same, the hypothesis test based on this permutation scheme has the correct size.

### Testing for differential connectivity of a class of genes

In a supervised analysis, we may be interested in knowing whether the network structure of a specific class of "interesting" genes ℱ, say, those corresponding to a particular biological function, has changed from one network to another. In an unsupervised ℱ, this could be a filtered subset of all genes , say those exhibiting at least five fold changes between the control and treatment samples. Another choice for ℱ could be a module for one of the networks.

We measure the average differential connectivity of gene pairs in ℱ between two networks by the following mean absolute distance (MDA) statistic

where  and  are the interaction scores between gene pair (*i*, *j*) in networks 1 and 2, respectively, with each constructed as in (2) using the gene expression data for that particular network. In using this measure as a test statistic, network connections of ℱ will be considered to be significantly different in the two networks if the value of Δ(ℱ) is sufficiently large. The measure is based on the widely-used *L*_1 _distance. Although there is no optimality theory in the general setting considered in this paper, this measure compared favorably with other distance- and entropy-based measures considered.

The p-value corresponding to Δ(ℱ) can be computed via random permutations as before

where the sum is taken over *P *randomly selected permutations *π *and

### Testing for differential connectivity of a single gene

The difference in connectivity of a single gene *g *in two networks can be assessed by the following MDA statistic

where the sum is over all remaining genes in a network and where  is the connectivity score between gene pair (*g*, *g'*) in networks *k *= 1, 2. The p-values for this statistic for each gene can be computed by permuting the pooled data columns and reconstructing the two networks using the permuted data followed by computation of this statistic for each pair of networks. Note that the p-values of all the genes can be computed simultaneously using the same set of random permutations.

## Authors' contributions

RG performed the computations and developed some of the methods. SoD developed most of the methods and planned the manuscript. SuD planned the study and provided the biological commentary. All three authors contributed towards the writing of the manuscript. All authors read and approved the final manuscript.
